# Biopsy-diagnosed renal granuloma after intravesical bacillus Calmette-Guérin therapy for bladder carcinoma: a case series and review of the literature

**DOI:** 10.1259/bjrcr.20190012

**Published:** 2019-11-15

**Authors:** Keiichi Narita, Hirotaka Akita, Eiji Kikuchi, Tadaki Nakahara, Shigeo Okuda, Seishi Nakatsuka, Mototsugu Oya, Masahiro Jinzaki

**Affiliations:** 1 Department of Radiology, Keio University School of Medicine, Shinjuku, Tokyo, Japan; 2 Department of Urology, Keio University School of Medicine, Shinjuku, Tokyo, Japan

## Abstract

Renal granuloma is a rare complication affecting the kidneys after intravesical bacillus Calmette-Guérin (BCG) therapy for bladder carcinoma. Our case series review describes the imaging and histopathological findings of BCG-induced renal granulomas. All three renal granulomas, which were located in the upper pole, had a solitary mass-like appearance. The mean diameter was 31.3 mm. In the two cases, the lesion was homogeneously enhanced on post-contrast CT, and presented homogeneous low signal intensity on *T*_2_ weighted imaging (*T*_2_WI) and iso-signal intensity on diffusion-weighted imaging (DWI). Both lesions had increased fludeoxyglucose (FDG) uptake. Histological examinations revealed granulomatous inflammation with fibrosis. The third case showed a lesion having heterogeneous enhancement on CT, heterogeneous and slightly high signal intensity on *T*_2_WI, and high signal intensity on DWI. This case showed more severe inflammatory cell infiltration and less fibrosis than the former two cases did. It was suggested that the signal intensity on *T*_2_WI and DWI depends on the degree of inflammation and fibrosis in renal granuloma. It is currently challenging to distinguish renal granuloma from renal malignancy based on only imaging findings. Biopsies were helpful in confirming the diagnosis and avoiding unnecessary resection. Renal granuloma should be considered as a differential diagnosis when a renal mass is found in a patient with a history of intravesical BCG treatment.

## Introduction

Intravesical administration of the bacillus Calmette-Guérin (BCG) vaccine for the treatment of superficial bladder carcinoma has become a mainstay of adjunctive therapy since the 1970s.^1^ Intravesical BCG therapy is a highly effective treatment for reducing the risk of recurrence and progression in patients with stage Ta tumors of high grade, carcinoma *in situ*, or Stage T1 tumors.^2^ Although the BCG vaccine is generally safe and tolerated by most patients, some local and systemic complications have been reported. Common local complications include cystitis, bladder contracture, granulomatous prostatitis, epididymo-orchitis, and ureteral obstruction, accompanied by symptoms and signs such as urinary frequency, dysuria, hematuria, malaise, and low-grade fever.^3,4^

Renal complications after intravesical BCG therapy include granulomatous lesion, abscess, pyelonephritis, and interstitial nephritis, which are very rare.^3,5,6^ Renal granulomas sometimes appear as expansile and mass-like lesions mimicking renal neoplasms.^7^ As a result, patients with renal granuloma might undergo unnecessary surgery.^8^ Thus, the differentiation between renal tumors and BCG-induced renal granulomas based on imaging findings is clinically important. Some authors have described the imaging findings of renal granuloma after BCG treatment as hypoenhancing on post-contrast CT^7–10^ and high signal intensity on *T*_2_ weighted imaging (*T*_2_WI).^8^ Senés et al reported the “central unaffected calyx sign,” which might be useful to differentiate renal granuloma from a malignant tumor.^10^ Normal calyx remains in the center of renal granuloma and is visible on post-contrast CT, while a malignant tumor tends to displace or destroy adjacent calyces. However, a comprehensive description of the imaging features and radiopathological correlation using various imaging modalities is lacking due to the rarity of the entity. Here, we present three cases of biopsy-diagnosed renal granuloma after BCG therapy.

## Case 1

**Figure 1.**
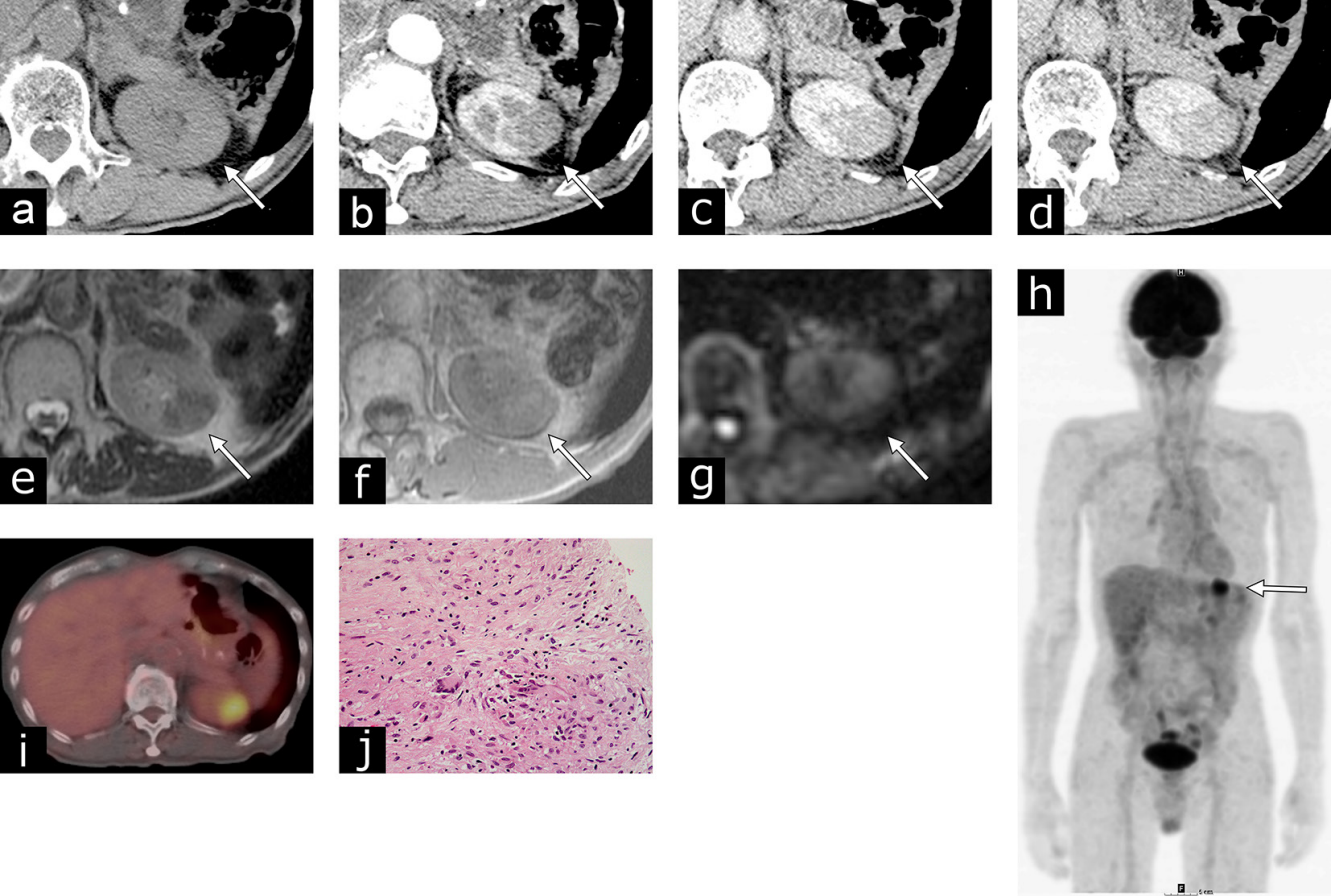
Case 1: Renal granuloma developing after intravesical bacillus Calmette-Guérin treatment for bladder carcinoma in a 73-year-old male. (a) An axial unenhanced CT image revealing an iso-attenuating mass (37 HU), compared to the renal parenchyma. (b–d) Dynamic contrast-enhanced CT findings showed a gradually enhanced renal mass. The CT attenuation value was 47 HU during the corticomedullary phase (b), 87 HU during the nephrographic phase (c), and 90 HU during the early excretory phase (d). (e) An axial *T*_2_ weighted image presenting low signal intensity for the renal lesion on the left kidney. (f) An axial *T*_1_ weighted image showing iso-signal intensity. (g) On an axial diffusion-weighted image, the lesion had iso-signal intensity. (h) A maximum intensity projection PET coronal image and (i) a PET/CT fusion image exhibiting focal FDG uptake at the upper pole of the left kidney with a SUV max of 9.4. (j) Hematoxylin and eosin staining indicating an epithelioid granuloma and fibrosis with multinucleated giant cells. FDG,fludeoxyglucose; HU, Hounsfield unit; SUV, standardized uptake value

A 73-year-old male presented with gross hematuria. A cystoscopy revealed a papillary tumor measuring approximately 2 cm on the left lateral bladder wall next to the ureteral orifice. Transurethral resection of the bladder tumor (TURBT) revealed a pTa high-grade urothelial carcinoma. He received intravesical BCG therapy (six weekly instillations of ImmuCyst at a dose of 81 mg). He did not experience lower urinary tract or systemic symptoms during the follow-up period. 3 months later, the urine cytology findings were negative, and repeated TURBT did not indicate any residual urothelial carcinoma. A 6 month follow-up CT revealed a renal mass at the upper pole of the left kidney (Figure 1). The size of the mass was 28 × 20 mm. The renal lesion appeared to be iso-attenuating [37 Hounsfield units (HU)], compared to the renal parenchyma, on unenhanced CT images. A dynamic contrast-enhanced CT scan revealed homogeneous and gradual enhancement. An MRI scan resulted in low signal intensity on *T*_2_WI, and iso-signal intensity on diffusion-weighted imaging (DWI). Pseudocapsules, calcifications, fat, and hemorrhagic components were not observed on the CT or MRI scans. The renal lesion exhibited increased fludeoxyglucose (FDG) uptake with a max standardized uptake value (SUV) of 9.4. We suspected renal granuloma based on his history of intravesical BCG therapy. An ultrasound-guided biopsy using an 18-gauge needle was performed, and the left renal lesion was histopathologically diagnosed as granulomatous inflammation with fibrosis, consistent with BCG granuloma. The patient was treated with antituberculous drugs, and the renal lesion gradually reduced in size and had disappeared after 43 months.

## Case 2

**Figure 2.**
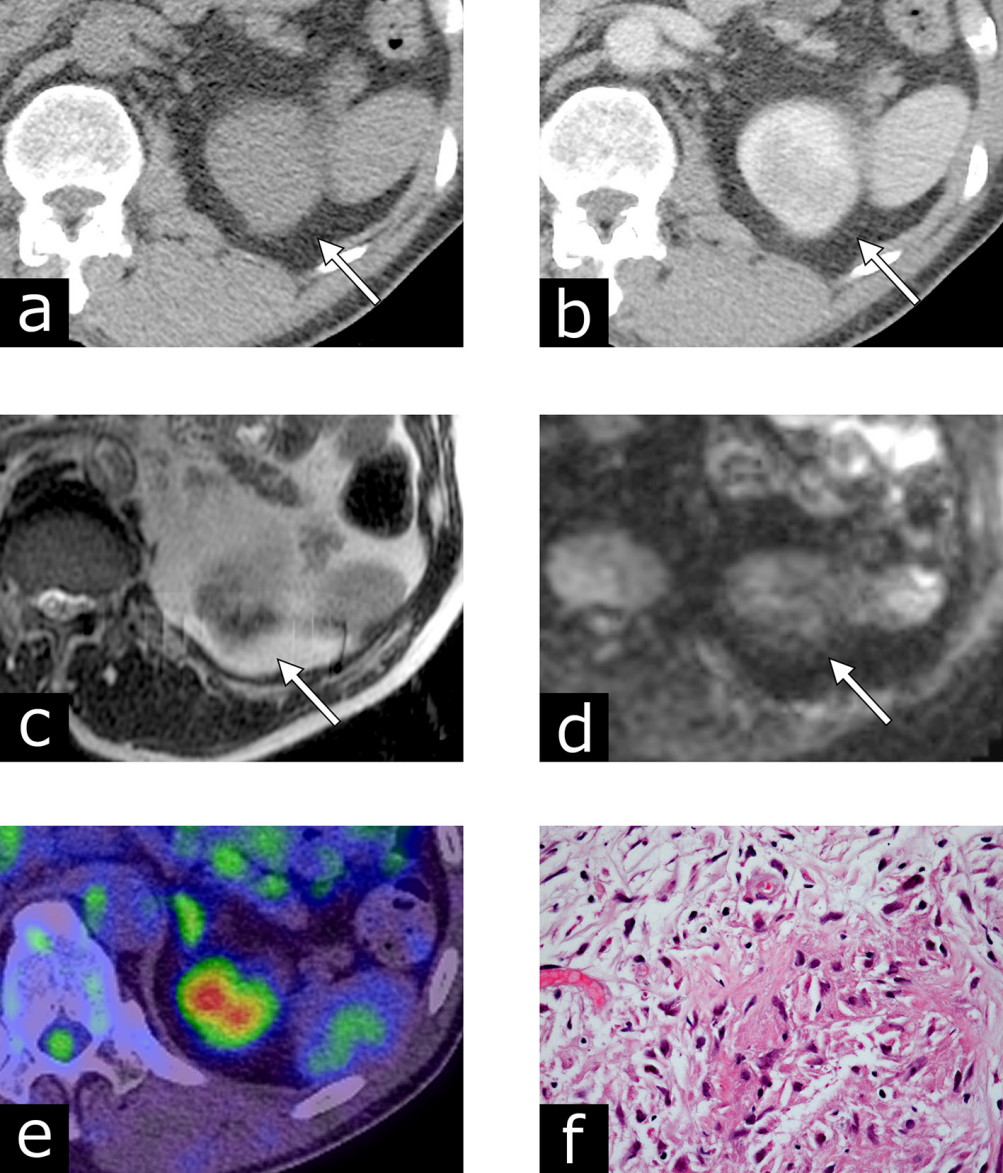
Case 2: Renal granuloma after intravesical bacillus Calmette-Guérin therapy for bladder carcinoma in a 67-year-old male. (a) An axial unenhanced CT image showing an iso-attenuating mass, compared to the renal parenchyma. (b) Contrast-enhanced CT revealing a homogenously enhanced renal mass. (c) An axial *T*_2_ weighted image presenting low signal intensity in the left renal lesion. (d) On the axial diffusion-weighted image, the lesion exhibits iso-signal intensity. (e) A PET/CT fusion image indicating increased FDG uptake at the lesion, with a maximum standardized uptake value of 6.4. (f) Hematoxylin and eosin staining demonstrating epithelioid granuloma and fibrosis with giant cells. FDG, fludeoxyglucose

A 67-year-old male, with a history of TURBT for pT1 solitary urothelial carcinoma 1.5 years prior, had suspicious urine cytology (Class III) on regular surveillance and exhibited wall thickening near the left ureteral orifice on CT imaging. TURBT revealed pT1 high-grade urothelial carcinoma. He received intravesical BCG therapy (six weekly instillations of ImmuCyst at a dose of 81 mg). He did not experience any lower urinary tract symptoms or systemic symptoms during the follow-up period. 3 months later, a follow-up CT revealed a renal mass measuring 39 × 30 mm at the upper pole of the left kidney (Figure 2). On an unenhanced CT image, the left renal lesion exhibited iso-attenuation (38 HU) compared to the renal parenchyma. A post-contrast CT scan revealed homogeneous enhancement. An MRI scan revealed low signal intensity on *T*_2_WI, and iso-signal intensity on DWI. Pseudocapsules, calcifications, fat, and hemorrhagic components were not detected on CT or MRI. PET/CT images indicated increased FDG uptake with a SUV max of 6.4 in the left renal lesion. Although renal granuloma was suspected from his history of intravesical BCG therapy, he underwent CT-guided biopsy using an 18-gauge needle to exclude renal malignancy. Histopathology revealed granulomatous inflammation with fibrosis, which is consistent with BCG granuloma. The patient was treated with antituberculous drugs, and the renal lesion had disappeared on CT after 9 months.

## Case 3

**Figure 3.**
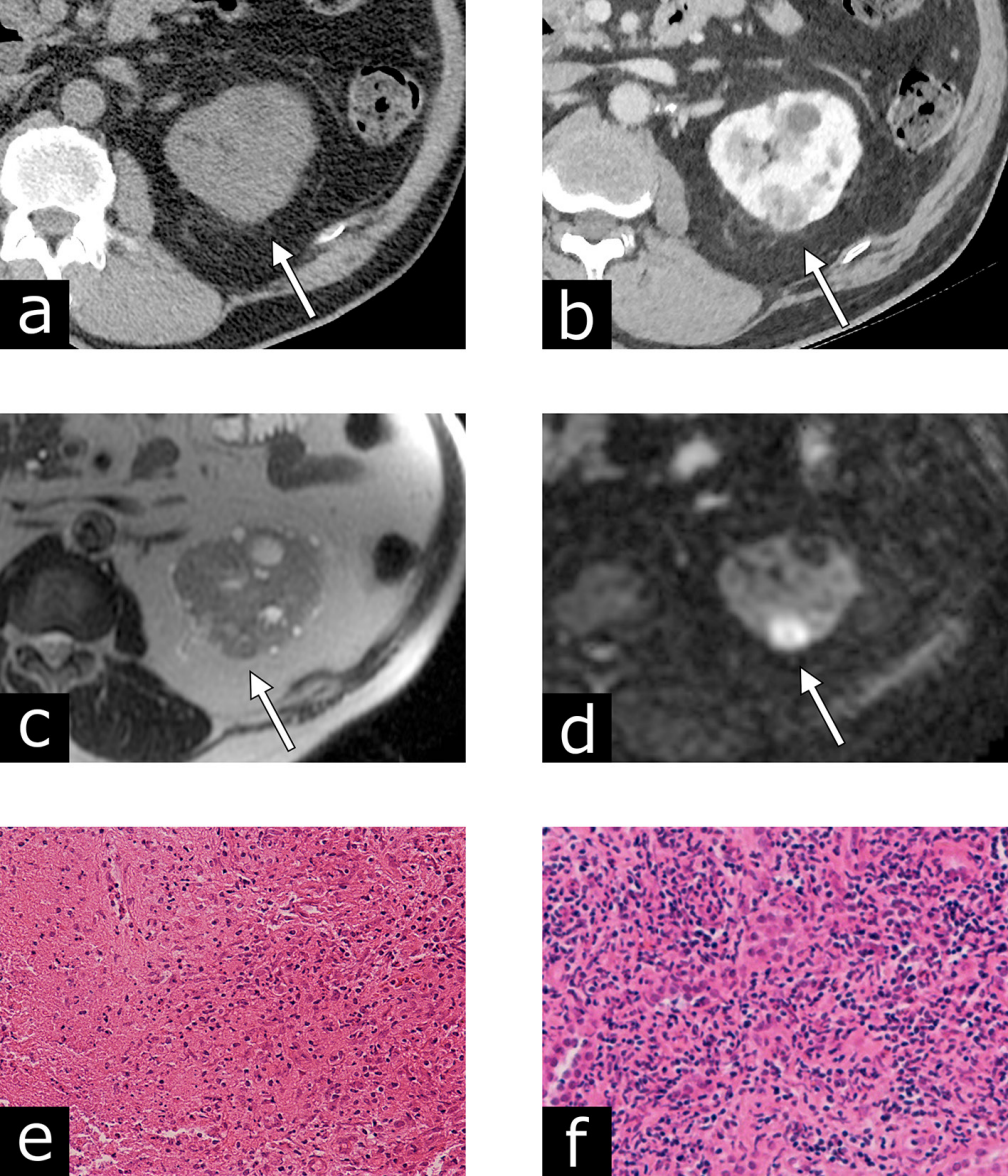
Case 3: Renal granuloma after intravesical bacillus Calmette-Guérin therapy for bladder carcinoma in a 71-year-old male. (a) An axial unenhanced CT image showing an iso-attenuating mass, compared to the renal parenchyma. (b) Contrast-enhanced CT revealing a heterogeneously enhanced renal mass during the nephrographic phase. (c) An axial *T*_2_ weighted image exhibiting heterogeneous and slightly high signal intensity in the left renal lesion. (d) An axial diffusion-weighted image indicating high signal intensity. The pathological specimen [hematoxylin–eosin staining: (e) low-power view, (f) high-power view] provides evidence of a necrotizing granuloma with severe infiltration of macrophages and lymphocytes

A 73-year-old male presented with gross hematuria. A cystoscopy revealed a sessile bladder tumor beside the right ureteral orifice. He underwent TURBT for pT1 high-grade urothelial carcinoma. He received initial intravesical BCG therapy (six weekly instillations of ImmuCyst at a dose of 81 mg) and a maintenance BCG instillation every 3 months. There was no recurrence of urothelial carcinoma as determined by regular surveillance during the 24 month follow-up period. At 28 months after the initial BCG therapy, a renal mass on the left kidney, which had not been present on the previous CT examination, was detected on follow-up CT (Figure 3). The renal mass at the upper pole of the left kidney measured 27 × 21 mm. The left renal mass exhibited iso-attenuation (28 HU) compared to the renal parenchyma on an unenhanced CT, and heterogeneous enhancement on a contrast-enhanced CT. An MRI scan revealed heterogeneous, slightly high signal intensity on *T*_2_WI, and high signal intensity on DWI. Pseudocapsules, calcifications, fat, and hemorrhagic components were not detected on CT or MRI. An ultrasound-guided 18-gauge needle biopsy of the left renal lesion indicated a necrotizing granuloma with inflammatory cell infiltration. The patient was managed conservatively without any antituberculous agent. The renal lesion subsequently decreased in size and had disappeared after 35 months. No recurrence of the renal lesion was observed during the follow-up period.

## Discussion

Renal granuloma after intravesical BCG therapy is a very rare complication. Lamm et al reported that renal abscesses arising as complications of BCG therapy occur in approximately 0.1% of patients receiving the BCG vaccine; however, the incidence of renal granuloma was not mentioned.^4^

The imaging findings for the three cases are summarized in Table 1. All three lesions had a solitary mass-like appearance, although multiple renal granulomas have been previously reported.^7,9–13^ All three renal lesions were located in the upper pole and likely developed from a retrograde infection via the ureter, although two mechanisms for the development of renal granuloma after BCG therapy have been suggested: direct seeding by vesicoureteral reflux, and hematogenous dissemination.^10^ Renal scars caused by vesicoureteral reflux are frequently observed in the upper poles because the upper poles tend to be affected by the intrarenal reflux of urine.^14^ On the other hand, hematogenous spread of BCG affects distant organs such as the lung, liver, and soft tissue. In our three cases, there were no findings suggesting tuberculosis except those in the kidney. Hence, vesicoureteral reflux was probably the route of infection in our three cases.

**Table 1.** Imaging features of three cases of renal granuloma after intravesical BCG treatment

Case	Age(y)/ Gender	Laterality	Location	Size (mm)	CT attenuation value (HU)				Homogeneity on NP	SI on	SI on DWI	FDG uptake	Period after the start of BCG therapy (months)
					Un	CMP	NP	EEP					
1	73 M	Left	Upper	28 × 20	37	47	87	90	homogenous	Low	Iso	Yes	6
2	67 M	Left	Upper	39 × 30	38	NA	113	NA	homogenous	Low	Iso	Yes	3
3	71 M	Left	Upper	27 × 21	28	NA	94	NA	heterogeneous	Slightly high, heterogeneous	High	NA	28

BCG = Bacillus Calmette-Guérin, *M* = male, HU = Hounsfield units, Un = unenhanced, CMP = corticomedullary phase, NP = nephrographic phase, EEP = early excretory phase, SI = signal intensity, T2WI = *T*_2_-weighted2-weighted images, DWI = diffusion-weighted images, NA = not available

Our cases exhibited two imaging patterns. One pattern (Cases 1 and 2) consisted of homogeneous enhancement on CT images, homogeneous and low signal intensity on *T*_2_WI, iso-intensity on DWI, and predominant fibrosis in the pathological specimens. Tuberculous granulomas typically exhibit a low signal intensity on *T*_2_WI, likely due to fibrosis and the release of free radicals from macrophages.^15–17^ The gradual enhancement observed during dynamic CT imaging was also thought to reflect fibrotic tissue.^18,19^ The other pattern (Case 3) consisted of heterogeneous enhancement on CT images, heterogeneous and slightly high signal intensity on *T*_2_WI, high intensity on DWI, and severe inflammation and less fibrosis in the pathological specimens. These findings support the previous report that the signal intensity on *T*_2_WI depends on the amount of macrophages, fibrosis, and cellular infiltrates in renal granuloma.^15^

To our knowledge, this is the first report that evaluates the DWI and FDG PET imaging characteristics of renal granulomas induced by BCG therapy. Granulomatous lesions are known to often exhibit FDG uptake as high as malignant tumors because of their increased metabolic activities.^20–23^ Regarding renal tumors, high-grade clear cell renal cell carcinomas (RCCs) (maximum standardized uptake value (SUV_max_), 6.2 ± 4.9) and papillary RCCs (SUV_max_, 5.9 ± 2.9) have higher SUVs than that of the normal renal parenchyma (SUV_max_, 2.2 ± 0.3), while low-grade clear cell RCCs, chromophobe RCCs, angiomyolipomas, and oncocytomas do not.^24^ Therefore, our cases suggest that differential diagnoses of hypermetabolic renal masses should include renal granuloma as well as high-grade clear cell RCC and papillary RCC.

Thus, differentiating renal granulomas and malignant renal tumors based only on imaging findings is likely to be challenging. Indeed, the findings of a homogeneous and gradual enhancement pattern on CT and a low *T*_2_WI signal intensity are similar to those of papillary RCC.^25,26^ Meanwhile, heterogeneous enhancement on CT findings, heterogeneous and slightly high signal intensity on *T*_2_WI findings, and high intensity on DWI are frequently observed in RCCs.^26,27^ The increased uptake of ^18^F-FDG is also observed in both BCG-related renal granulomas and RCCs, as mentioned above. Accordingly, when a patient has a history of intravesical BCG therapy for bladder cancer, the possibility of BCG-related renal granuloma should be considered as a differential diagnosis.

In Case 3, the renal granuloma appeared 28 months after the start of BCG therapy. Although the majority of side-effects occurs within the initial 6 months after treatment, some local and systemic side-effects have been observed during the 1–3 years of maintenance BCG treatment.^28,29^ Thus, the possibility of a renal granuloma should be considered even if the lesion appears more than 6 months after the start of BCG therapy.

For the final diagnosis and the determination of a treatment strategy for BCG-related renal granuloma, a needle biopsy is useful, as was demonstrated in our three cases. After treatment with antituberculous drugs in two cases and conservative follow-up in one case, all three lesions decreased in size and eventually disappeared. Three previous papers reported the use of a needle biopsy for a final confirmation and concluded that a biopsy could exclude the possibility of primary or metastatic malignant tumors, avoiding unnecessary surgical resection.^9,10,13^ Most of the previous cases were treated with antituberculous drugs,^7,8,10,13,30,31^ and Green DB et al reported that corticosteroids and antimycobacterial agents are the preferred treatments for renal granuloma.^32^ However, Al-Qaoud T et al reported two patients who were managed without antituberculous medications and concluded that asymptomatic renal granulomas may resolve spontaneously without the use of antituberculous agents.^9^

In conclusion, we have reported the imaging characteristics of renal granulomas after intravesical BCG therapy. The lesions appeared to be iso-attenuating on unenhanced CT images and were moderately enhanced after intravenous contrast injection. The signal intensity on *T*_2_WI and DWI varied, likely depending on the degree of inflammation and fibrosis. The lesions exhibited increased FDG uptake; their SUVs were within the same range as those reported for RCCs. BCG-related renal granuloma should be considered as a differential diagnosis when interpreting the imaging features of a renal mass in a patient with a history of intravesical BCG therapy for bladder carcinoma. Renal granuloma could appear a few years after the start of BCG therapy. A biopsy was helpful in obtaining a correct diagnosis and excluding the possibility of a malignant tumor.

## Consent

Informed consent could not be obtained despite exhaustive attempts. However, patient anonymity has been maintained in all cases.

## Learning points

1. Renal granuloma is a rare side-effect after intravesical BCG therapy for bladder carcinoma.
2. The BCG-induced renal granulomas exhibited moderate enhancement on CT and increased FDG uptake. The signal intensity on *T*_2_WI and DWI varied, likely depending on the degree of inflammation.
3. A needle biopsy was helpful in confirming the diagnosis and excluding malignancy.
